# Salvianolic acid A regulates pyroptosis of endothelial cells *via* directly targeting PKM2 and ameliorates diabetic atherosclerosis

**DOI:** 10.3389/fphar.2022.1009229

**Published:** 2022-11-08

**Authors:** Ji Zhu, Hang Chen, Yifei Le, Jianan Guo, Zhijun Liu, Xiaobing Dou, Dezhao Lu

**Affiliations:** ^1^ The Third School of Clinical Medicine (School of Rehabilitation Medicine), Zhejiang Chinese Medical University, Hangzhou, China; ^2^ The Third Affiliated Hospital of Zhejiang Chinese Medical University, Hangzhou, China; ^3^ School of Life Sciences, Zhejiang Chinese Medical University, Hangzhou, China

**Keywords:** Salvianolic acid A, pyroptosis, endothelial cells, PKM2, diabetic atherosclerosis

## Abstract

Rescuing endothelial cells from pyroptotic cell death emerges as a potential therapeutic strategy to combat diabetic atherosclerosis. Salvianolic acid A (SAA) is a major water-soluble phenolic acid in the *Salvia miltiorrhiza* Bunge, which has been used in traditional Chinese medicine (TCM) and health food products for a long time. This study investigated whether SAA-regulated pyruvate kinase M2 (PKM2) functions to protect endothelial cells. In streptozotocin (STZ)-induced diabetic ApoE^−/−^ mice subjected to a Western diet, SAA attenuated atherosclerotic plaque formation and inhibited pathological changes in the aorta. In addition, SAA significantly prevented NLRP3 inflammasome activation and pyroptosis of endothelial cells in the diabetic atherosclerotic aortic sinus or those exposed to high glucose. Mechanistically, PKM2 was verified to be the main target of SAA. We further revealed that SAA directly interacts with PKM2 at its activator pocket, inhibits phosphorylation of Y105, and hinders the nuclear translocation of PKM2. Also, SAA consistently decreased high glucose-induced overproduction of lactate and partially lactate-dependent phosphorylation of PKR (a regulator of the NLRP3 inflammasome). Further assay on Phenylalanine (PKM2 activity inhibitor) proved that SAA exhibits the function in high glucose-induced pyroptosis of endothelial cells dependently on PKM2 regulation. Furthermore, an assay on c16 (inhibitor of PKR activity) with co-phenylalanine demonstrated that the regulation of the phosphorylated PKR partially drives PKM2-dependent SAA modulation of cell pyroptosis. Therefore, this article reports on the novel function of SAA in the pyroptosis of endothelial cells and diabetic atherosclerosis, which provides important insights into immunometabolism reprogramming that is important for diabetic cardiovascular disease complications therapy.

## Introduction

Diabetic cardiovascular disease complications, the major cause of mortality among people with type 2 diabetes mellitus (T2DM), affect approximately 32.2% of all persons with T2DM (Einarson et al., 2018). Although various pathological factors contribute to atherosclerotic coronary artery disease, diabetes becomes the primary risk factor associated with the high incidence of atherosclerotic coronary artery disease (Davidoff et al., 2004; Balakumar et al., 2016). In individuals with diabetes mellitus (DM), the risk of developing coronary heart disease is two to six times greater than those without DM(Martín-Timón, 2014). Atherosclerosis is a chronic inflammatory disease (Ross, 1999), whereby unrelenting inflammatory response and cell death are thought to be the main drivers of atherosclerosis development (Engelen et al., 2022). Recently, it has been found that T2DM provokes multiple inflammatory factors at low concentrations promoting chronic low-grade inflammatory responses. This inflammatory mechanism is currently considered a leading mechanism for the development and progression of diabetic atherosclerosis (Marfella et al., 2007). Located at the interface between blood and interstitial tissue, the endothelium forms a protective barrier against endogenous danger signals (Roumenina et al., 2016). Endothelial cells are the first line of defense against inflammatory damage in the vascular system (Valbuena and Walker, 2006), and the death of these cells usually signifies a critical and initial stage in the development of atherosclerosis (Nawa et al., 2002). It is believed that hyperglycemia-induced endothelial cell inflammation is a recognized cause of vascular complications in T2DM (Knapp et al., 2019).

It is believed that pyroptosis represents a pattern of inflammatory programmed death and causes alterations to the ultrastructure of the cardiovascular system, which leads to further significant damage and a poor prognosis for cardiovascular disease (Patel et al., 2017). Increasing evidence has highlighted an essential role of pyroptosis in the modulation of endothelial damage and compromised the stability of arterial wall plaque, angiogenesis, inflammatory infiltration, and smooth muscle cell hyperplasia, suggesting a critical role of pyroptosis in atherosclerosis (Wang et al., 2020). Pyroptosis is the primary type of atherosclerosis-associated endothelial cell death, which occurs in endothelial cells and starts at the early stages of atherosclerosis and is associated with endothelial activation, monocyte recruitment, and atherosclerosis formation (He et al., 2021). Both infectious and non-infectious stimuli can trigger pyroptosis, and subsequent studies of chronic metabolic diseases revealed that excess nutrients in the organism, including glucose, cholesterol, lipids, etc., are also the causative factors of pyroptosis (Xue et al., 2019). Some evidence shows that a high glucose level causes endothelial cell pyroptosis in the progression of various diseases (Song et al., 2019; Kong et al., 2022). Therefore, inhibition of endothelial cell pyroptosis may prevent cardiovascular diseases in diabetic individuals.

Excessive activation of glycolysis has previously been associated with endothelial cell inflammation, dysfunction, and proliferation (Feng et al., 2017; Wu et al., 2017). Maintaining the metabolic homeostasis of endothelial cells by adjusting glycolysis to reduce dysfunction and inflammation could represent a novel therapeutic strategy for AS (Li et al., 2019a). Several key enzymes in glycolysis may provide insights for understanding the association between endothelial cell injury and AS (Yang et al., 2018; Perrotta et al., 2022). Several glycolysis-related proteins highly expressed in atherosclerotic plaques and positively correlated with AS development have been identified (Sluimer et al., 2008; Tawakol et al., 2015; Lü et al., 2018; Doddapattar et al., 2022). Recent studies have found that the high glucose level activates classical pathways that promote endothelial pyroptotic cell death *in vivo* and vitro (Song et al., 2019; Kong et al., 2022). The high glucose level drives precursors of caspase-1 and pattern recognition receptors, e.g., NLRP3, to form a macromolecular complex, i.e., inflammasome, *via* the junctional protein, ASC. Activated caspase-1 (a form of caspase-1 cleaved by NLRP3-inflammasome) then cleaves GasderminD (GSDMD), thus forming N and C termini of GSDMD. The binding of GSDMD with the phospholipid proteins on the cellular membrane at the N terminus results in the formation of pores, allowing the contents to be released into the cells to induce pyroptosis. Concurrently, the activated Caspase-1 cleaves the precursors of IL-1β and IL-18, resulting in the formation of active IL-1β and IL-18, which are released into the extracellular space to induce a micro-environmental inflammatory response (Shi et al., 2017). Evidence suggests that endothelial pyroptosis is highly associated with increased glycolysis (Jin et al., 2021). One of the glycolysis-related proteins that are positively correlated with AS development is called pyruvate kinase type M2 (PKM2), which activates the assembly of inflammasome element, PKR (known as EIF2AK2), in a lactate-dependent phosphorylated-form to facilitate the formation of the inflammasome by NLRP3 and ASC(Shirai et al., 2016; Xie et al., 2016). PKM2 has also been implicated as a key regulator in various diabetes-related diseases (Qi et al., 2017; Srivastava et al., 2018). Additionally, PKM2 has been associated with activating NLRP3 inflammasome of blood macrophage in diabetic patients and is positively correlated with atherosclerotic plaque susceptibility (Li et al., 2020).

*Danshen* (*Salvia miltiorrhiza* root) is widely used as a health food and as a Traditional Chinese Medicine, which has been associated with multiple properties, particularly in the prevention and treatment of cardiovascular and metabolic diseases. Salvianolic acid A (SAA) is the main water-soluble and biologically active ingredient of *Danshen*. Several recent reports have demonstrated the anti-diabetic activity of SAA. It has been discovered that SAA inhibits the activation and aggregation of platelet in patients with type 2 diabetes mellitus (Zhou et al., 2020). Moreover, the reports have revealed that SAA has a protective effect on diabetic complications in multiple rodent models, such as inhibiting hepatic fibrosis in HFD-fed and STZ-induced type 2 diabetic rats (Qiang et al., 2014), attenuating kidney injury and inflammation in 5/6 nephrectomized rats (Zhang et al., 2018), ameliorating early-stage atherosclerosis development *via* NLRP3 inflammasome in Zucker diabetic fatty rats (Zhang et al., 2018), and improving diabetic peripheral neuropathy in KK-Ay mice (Xu et al., 2020a). The role of SAA in alleviating endothelial dysfunction of diabetic angiopathy and diabetic atherosclerosis in ApoE^−/−^ mice has yet to be verified. Thus, we sought to determine whether SAA could improve diabetic atherosclerosis in the ApoE^−/−^ mice with diabetes mellitus by inhibiting endothelial pyroptosis of aortic sinuses. Our results implied that SAA could ameliorate atherosclerosis in Western diet-fed STZ-induced diabetic ApoE^−/−^ mice by regulating endothelial pyroptosis *via* the PKM2/PKR/NLRP3 inflammasome signaling pathway, demonstrating the potential use of SAA as a therapy to relieve diabetes mellitus and its related macrovascular complications.

## Materials and methods

### Establishment of experimental animal models

Male ApoE^−/−^ mice (18–22 g, six weeks of age) were purchased from GemPharmatech Co., Ltd. (Nanjing, China). The mice were domesticated in a room with a 12 h light/dark cycle at constant room temperature for a minimum of seven days. The mice were allowed *ad libitum* access to standard rodent chow and water. All animal experimentations performed in this study were conducted according to the Guide of Chinese Regulation for the Use and Care of Laboratory Animals. The experiments were approved by the Medical Code and Ethics Committee of Zhejiang Chinese Medical University (approval number: 20200720-06).

**FIGURE 1:**
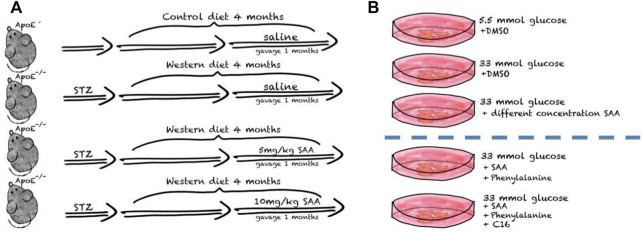
Schematic diagram of SAA treatments in diabetic AS mouse model.

After one week of environmental adaptation, 50 mg/kg streptozocin (STZ, in citrate buffer, Sigma-Aldrich, St Louis, MO, United States) daily was administered intraperitoneally to mice for 5 consecutive days to induce diabetes (Yi and Maeda, 2006; Tikellis et al., 2008). After hyperglycemia, i.e., blood glucose levels reached 16.7 mmol L^−1^ at 1 week post injection, both groups received a Western diet with 0.5% cholesterol (Trophi Feed High-tech Co., Ltd., Nantong, China) for 12 weeks. After establishing the AS model, ApoE^−/−^ male mice were randomly divided into three weight-matched groups: 1) the Western diet (WD) AS model group consistently received a Western diet for four weeks and intraperitoneal injections with normal saline daily; 2) the low SAA group consistently receiving Western diet for four weeks and orogastric gavage with 10 mg/kg SAA daily; 3) the high SAA group consistently receiving Western diet for four weeks and orogastric gavage with 20 mg/kg SAA daily. A group of 6 male ApoE^−/−^ mice were injected with an equal volume of citrate buffer (vehicle) and then fed with a normal diet was used as a control. And Salvianolic acid A was purchased from Herbpurify CO., LTD. (Chengdu, China) and Figure 1 is the schematic diagram of SAA treatments in diabetic AS mouse model.

### Histological section

Serial 8 μm-thick cryosections were collected continuously from the central ventricle to the aortic arch. The sections were stained with Oil-Red-O, a technique used to demonstrate fatty degeneration and abnormal lipid-like sedation. H&E and Masson staining determine the plaque area and collagen fiber content. Images were taken by a Digital pathological section scanning system (NanoZoomer C13210-01, Hamamatsu, Japan) and analyzed by the NDP.view2.

### Immunofluorescence

Frozen sections were first processed for antigen retrieval before staining. After blocking with 10% goat serum in PBS for 30 min at 37°C, the sections were incubated overnight with GSDMD (1:50, Santa Cruz, United States), Phospho-PKM2 (1:100, Affinity Biosciences, China), ASC (1:100, AdipoGen, United States), NLRP3 (1:100, AdipoGen, United States), CD31 (1:100, Abcam, United States) at 4°C. Next, the sections were incubated with fluorochrome-conjugated secondary antibodies. DAPI was used to counterstain the slides. For the Huvecs immunofluorescence, Huvecs were fixed with 4% paraformaldehyde for 15 min and permeabilized with 0.1% Triton X-100 for 10 min at room temperature. After blocking for 60 min at 37°C with 10% goat serum, Huvecs were incubated with the matching primary antibodies at 4°C overnight. Then, the sample was incubated with the corresponding secondary antibodies, and the signal was detected *via* visualization using a fluorescence microscope.

### Cell culture

HUVECs (Meisen Cell, CTCC-009-063, China) were cultured in Endothelial Cell Medium (ECM, ScienCell, cat. #1001) supplied with 5% fetal bovine in 5% CO_2_ at 37°C. Cells at 50% confluence were exposed to high glucose (33 mM) for 48 h to establish a pyroptosis model *in vitro*. Passage number of HUVECs in experiment was between 3 and 10.

### Bioinformatics analysis

The targets of SAA were predicted using SwissTargetPrediction (http://www.swisstargetprediction.ch/), Comparative Toxicogenomics Database (http://ctdbase.org/), and Traditional Chinese Medicine Systems Pharmacology Database and Analysis Platform (https://tcmsp-e.com/tcmsp.php). Potential targets related to ischemic stroke were predicted using DisGeNet (https://www.disgenet.org/search), a database for human genetic disease prediction (Piñero et al., 2017). Overlapping targets in both groups were analyzed by the jvenn tool (http://bioinfo.genotoul.fr/jvenn) and by String software (https://string-db. org/) to investigate the interaction network. Potential pathways were analyzed using the DAVID v6.8 tool (https://david.ncifcrf.gov/) with pathway enrichment from Gene Ontology (GO) and the Kyoto Encyclopedia of Genes and Genomes (KEGG). And statistical analyses were performed using the Sangerbox tools, an online platform for data analysis (http://www.sangerbox.com/tool) (Yu et al., 2012).

### Western blotting analysis

Total proteins were extracted from the HUVECs with RIPA Lysis containing protease and phosphatase inhibitor (Epizyme, China) for western blotting. The supernatant was collected, and equal amounts of protein (20 μg) were subjected to electrophoresis on 10% SDS-PAGE gels (Epizyme, China) following protein quantification using a BCA protein assay kit (Vazime, China). The protein was transferred to a polyvinylidene fluoride (PVDF) membrane (Millipore, United States) and blocked with 5% dry milk for 2 h at room temperature. The PVDF membranes were incubated with the specific primary antibodies to Phospho-PKM2 (1:1,000, YP1444, immunoway, China), PKM2 (1:20000, 60268-1-Ig, Proteintech, China), Phospho-PKR(1:1,000, RT1503, HUABIO, China), PKR(1:1,000, 18244-1-AP, Proteintech, China), NLRP3 (1:1,000, AG-20B-0014, AdipoGen, United States), ASC(1:1,000, AG-25B-0006, AdipoGen, United States), GSDMD (1:500, sc-393656, Santa Cruz, United States), cleaved N-terminal GSDMD (1:1,000, ab215203, Abcam, United Kingdom), Caspase-1 (1:1,000, ab179515, Abcam, United Kingdom), and *ß*-Actin (1:2000, 20536-1-AP, Proteintech, China) overnight at 4°C, followed by incubation with secondary antibodies (1:5,000, BOSTER Biological Technology, China). The membrane was exposed to an enhanced chemiluminescence kit (Vazime, China) and observed using a Clinx ChemiScope 3,500 (Clinx Science instrument Co. Ltd., China). Band density was calculated with the ImageJ software.

### Lactate production assays

Cellular lactate production was measured by lactate assay kit (Solarbio, China) according to the manufacturer’s instructions. All experiments were normalized by the cell number.

### Molecule-docking assay

The molecular docking of SAA to PKM2 (PDB code: 1T5A, http://www.rcsb.org/) was conducted in the Yinfo Cloud Platform (http://cloud.yinfotek.com/). AutoDock Vina (Trott and Olson, 2009) program was utilized to execute semi-flexible docking, and output poses were evaluated.

### Cellular thermal shift assay

HUVECs were lysed by liquid nitrogen, and the lysates were clarified by centrifugation at × 16,000 g for 15 min at 4°C, followed by the collection of the soluble fraction. The resultant cell lysates were divided into two fractions: one fraction was incubated with solvent and assigned as the control group, and another fraction was incubated with SAA (25 μmol/L) for 30 min at room temperature and assigned as the SAA-treated group. After incubation, 100 μl of lysates were aliquoted into PCR tubes. The lysates were then heated for 3 min in a gradient PCR machine at sequentially increased temperature (39–66°C with a temperature interval of 3°C).

For CETSA experiments on living cells, HUVECs were pre-treated with SAA (25 μmol/L) for 24 h and heated for 3 min at indicated temperatures, followed by cooling at room temperature. Next, the cells were collected, and 100 μl of protein loading buffer was added to the cells before boiling for 10 min. Then, lysates were assayed by immunoblotting against PKM2.

### Statistical analysis

GraphPad Prism 8.0 software was used for statistical analysis. After confirming that all the variables were normally distributed. Statistical significance was assessed using Student’s *t* test for two sample datasets. Where more than two samples were compared, statistical significance was assessed using one- or two-way analysis of variance (ANOVA) followed by Dunnet’s or Sidak’s multiple comparison tests, respectively. All tests were two-tailed. *p* < 0.05 was considered statistically significant.

## Results

### Salvianolic acid A attenuated the formation of atherosclerotic lesions in western diet-fed diabetic ApoE^−/−^ mice

**FIGURE 2:**
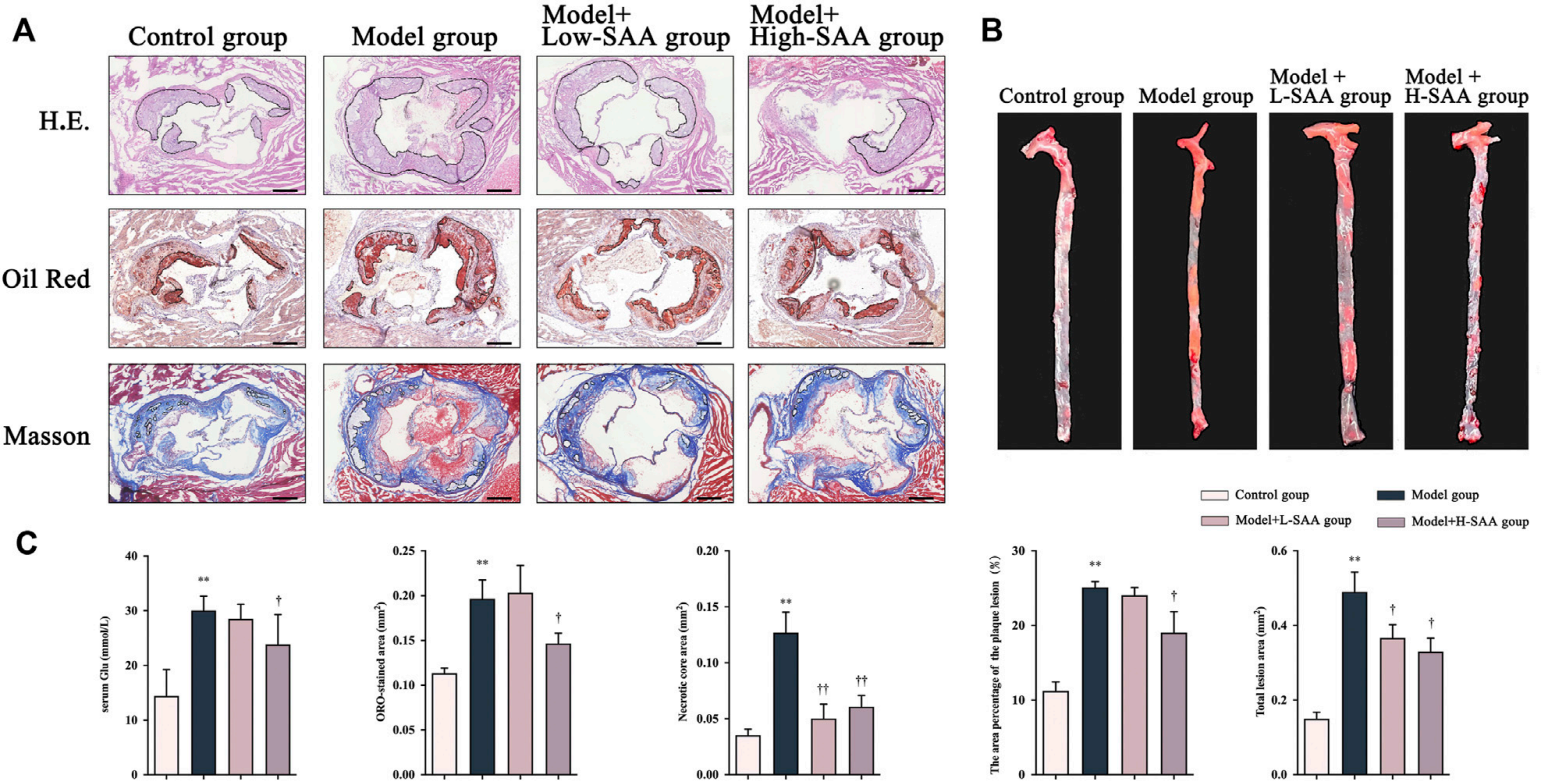
SAA attenuated the formation of atherosclerotic lesions in Western diet-fed diabetic ApoE^−/−^ mice. **(A)** Representative H.E. staining, oil red staining and Masson Trichrome staining of sectioned aortic roots (n = 4 ). **(B)** Representative oil red O staining of en face aorta (n = 4 ). **(C)** The content of serum glucose in mice.

To investigate the effect of SAA on diabetic atherosclerosis, ApoE^−/−^ mice were fed a Western diet for 12 weeks after STZ injection, followed by treatment with SAA for four weeks. The en-face aorta and cross-sectional view of the aortic sinus arch stained with Oil Red-O reflect the development of lipid plaques. The oil red O stain in *en face* aorta and aortic sinus lesions indicates the significant increase in the AS plaque of aorta in Western diet-fed diabetic ApoE^−/−^ mice compared with control diet-fed non-diabetic ApoE^−/−^ mice (Figures 2A,B). Compared with diabetic ApoE^−/−^ mice treated with saline, the diabetic ApoE^−/−^ mice treated with SAA exhibited a significant decrease in AS plaque area in *en face* aorta and aortic sinus in a dose-dependent (Figures 2A,B). These findings were further verified by detecting histopathological changes in the cross-sectional view of the aorta by HE staining. Diabetic ApoE^−/−^ mice fed with a Western diet appeared to have a greater area of plaque within the aortic sinus, more disorganized vessel wall structure with irregular local intimal rupture, higher levels of lipid deposition, and greater numbers of infiltrating foam cells than that of control diet-fed ApoE^−/−^ mice (Figure 2A).

Additionally, the diabetic ApoE^−/−^ mice treated with SAA demonstrated alleviation of histopathological changes, with fewer atherosclerotic plaques in the vessel walls than that of diabetic ApoE^−/−^ mice treated with saline. Masson staining of aortic cross-sectional views revealed that the Western diet-fed diabetic ApoE^−/−^ mice had less collagen fibril content, more cholesterol crystals, and larger necrotic cores than that control diet-fed ApoE^−/−^ mice (Figure 2A). Notably, the treatment with SAA has alleviated these pathological changes in the aorta of the mice (Figure 2). These findings demonstrate that SAA treatment decreased the atherosclerotic plaque formation and inhibited the pathological changes in the aorta of diabetic apoE^−/−^ mice.

### Network pharmacology analysis of Salvianolic acid A in diabetic atherosclerosis

**FIGURE 3:**
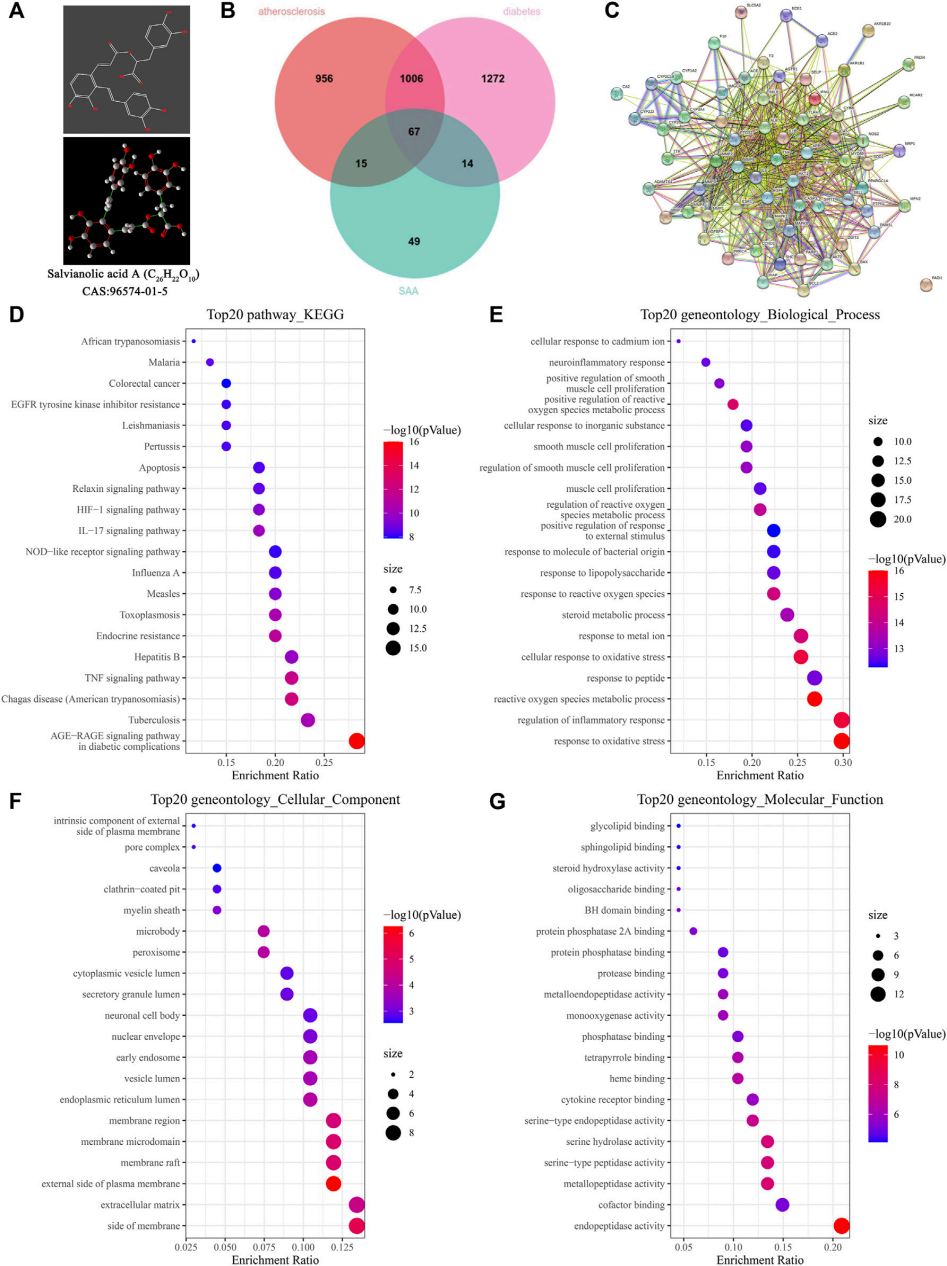
Network pharmacology analysis of SAA in diabetic atherosclerosis. **(A)** The chemical structure of SAA. **(B)** The overlapping targets of diabetic AS and SAA. **(C)** The interaction network of the overlapping targets. **(D)** KEGG enrichment analysis for the overlapping targets. **(E–G)** GO analysis for the overlapping targets.

The predicted targets were studied to explore the pharmacological mechanism of SAA in diabetic atherosclerosis. The chemical structure of SAA is presented in Figure 3A. The potential targets of SAA were predicted using databases (a total of 145 targets), and the genes related to diabetes and atherosclerosis were predicted using DisGeNet (a total of 1,073 targets). A total of 67 targets were overlapped by matching the two groups of targets (Figure 3B). The interaction networks of the 67 targets are illustrated in Figure 3C. These results suggest that SAA might regulate diabetic atherosclerosis through the action of the 67 overlapping targets. To explore the potential mechanism of SAA in diabetic atherosclerosis, the 67 overlapping targets were analyzed by GO annotations and KEGG enrichment analyses. Figures 3D–G shows that these targets are involved in immune and inflammatory responses, i.e., NOD-like receptor signaling pathway, TNF signaling pathway, etc. Thus, we deduce that SAA may influence the progression of diabetic atherosclerosis by regulating the inflammatory response pathway.

### Salvianolic acid Areduced endothelial cells of the aortic sinus pyroptosis in the western diet-fed diabetic ApoE^−/−^ mice

**FIGURE 4:**
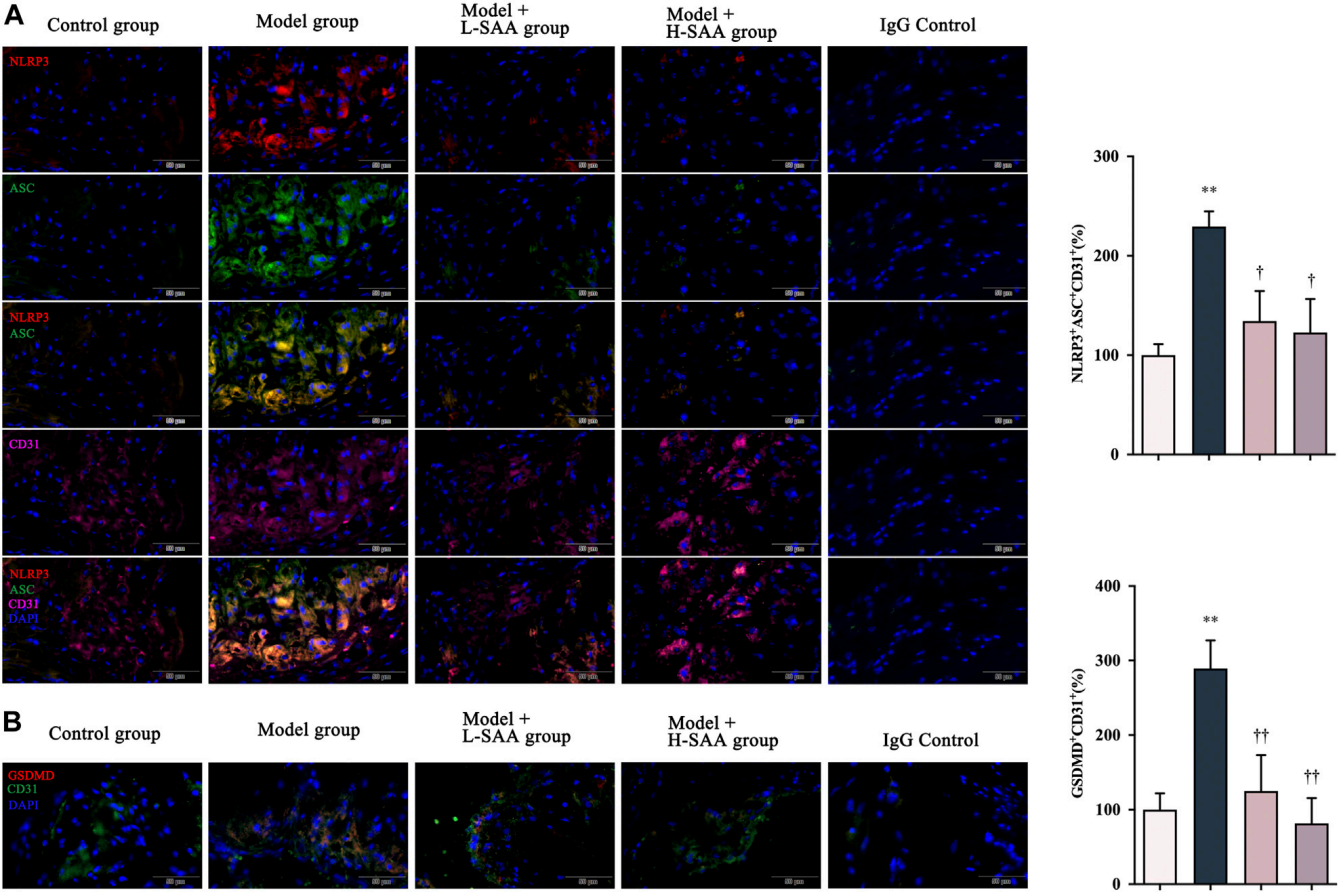
SAA reduced endothelial cells of the aortic sinus pyroptosis in the Western diet-fed diabetic ApoE^−/−^ mice. **(A)** NLRP3, ASC and CD31 levels in aortic roots were detected by immunofluorescence (*n* = 3). **(B)** GSDMD levels in aortic roots were detected by immunofluorescence (*n* = 3).

Pyroptosis is a form of pro-inflammatory programmed cell death induced by the NOD-like receptor signaling pathway. It has been found that endothelial cell pyrotposis contributes to atherosclerosis (He et al., 2021). To explore the protective roles of SAA on pyroptosis, we compared the expression of NLRP3, ASC, and GSDMD in the aortic sinus of all groups (Figure 4). Immunohistochemical fluorescence assay revealed that the levels of NLRP3, ASC, and GSDMD were significantly increased in the ApoE^−/−^ mice following induction of diabetes and consistent feeding with a Western diet compared with the ApoE^−/−^ mice fed with a control diet. However, this increasing trend was reversed considerably by SAA. Multiplex immunofluorescence staining was conducted to detect the expression and location of the critical molecules of pyroptosis, namely NLRP3 and ASC in vascular endothelium (CD31 marker) (Figure 4). The results revealed that NLRP3 and ASC are more co-localized with endothelial marker CD31 in the aortic sinus of the diabetic ApoE^−/−^ mice fed with a Western diet than that of ApoE^−/−^ mice from other groups. Furthermore, SAA decreased the expression of NLRP3 (displayed in AF594 and marked in red), ASC (demonstrated in AF488 and marked in green) in endothelial cells (CD31^+^, displayed in AF647 and marked in purple) in the diabetic AS mice in a dose-dependent manner.

### Salvianolic acid A Inhibited High Glucose-induced pyroptosis in HUVECs

**FIGURE 5:**
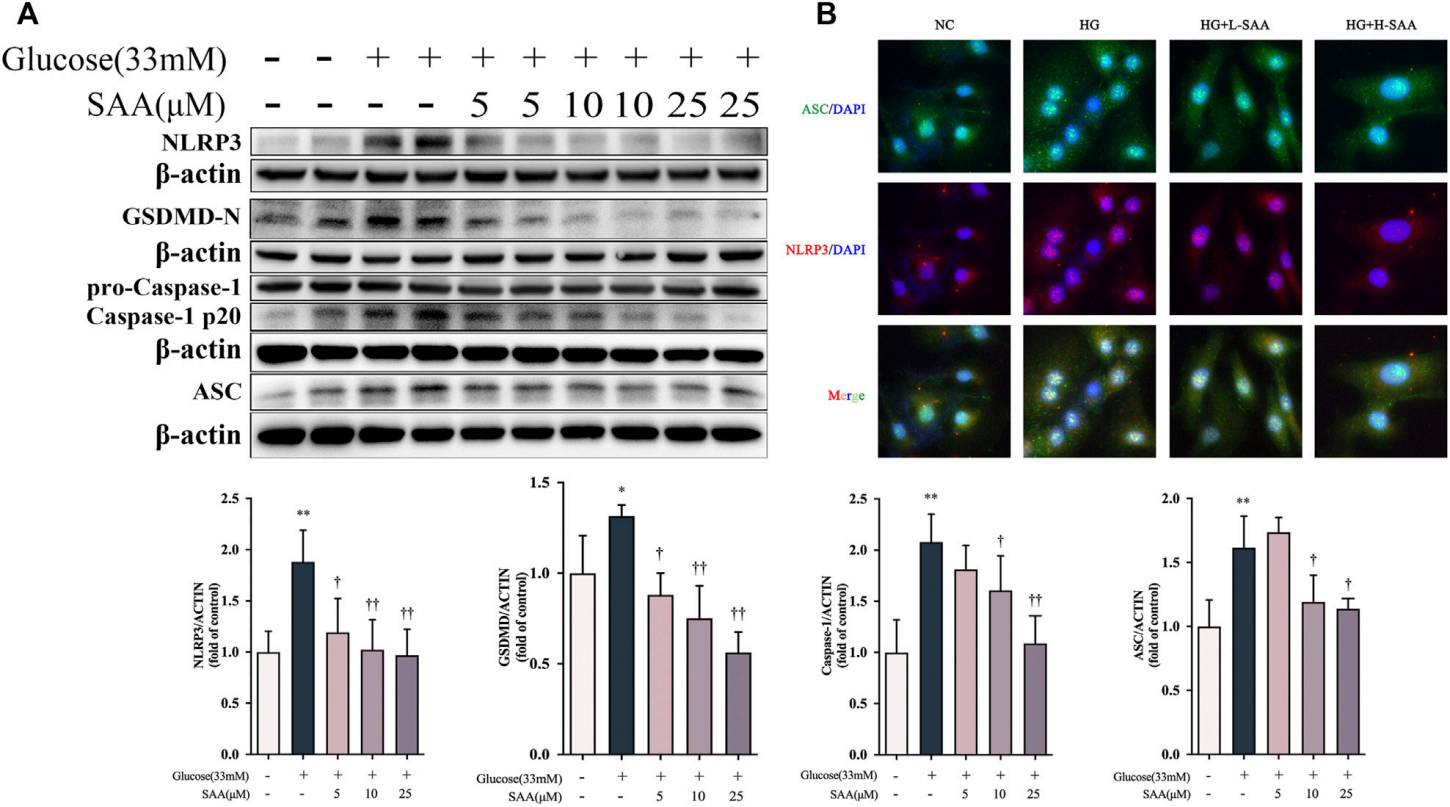
SAA inhibited high glucose-induced pyroptosis in HUVECs. **(A)** Expression levels of NLRP3, ASC, caspase-1, GSDMD and *ß*-Actin in HUVECs were determined by western blot. **(B)** Cellular localization of NLRP3 and ASC was analyzed by immunofluorescence staining. Data of western blot are the representatives of four independent experiments. Date of histograms are represented as mean ± SD of three independent experiments. *p < 0.05, **p < 0.01 vs. control group. ^†^ *p* < 0.05, ^††^ *p* < 0.01 vs. high glucose group.

We investigated whether a high glucose concentration could induce the expression of pyroptosis-related proteins in HUVECs. Western blotting results demonstrate that glucose, at the concentration of 33 mM, could promote the activation of the NLRP3 and cleavage of caspase-1 and GSDMD-N. At the same time, the treatment with SAA in a dose-dependent manner decreased the expression levels of these pyroptosis-related proteins (Figure 5A). And these concentrations of SAA can produce no damage to HUVECs (Supplementary Figure S1). We then detected the level of NLRP3-ASC oligomers by a fluorescence colocalization method. The results obtained from the fluorescence colocalization experiment are consistent with the results of Western blot, which indicate that at the concentration of 33 mM, glucose caused numerous foci of NLRP3 and ASC to form in HUVEC; a treatment with SAA was proven to alleviate this condition (Figure 5B). However, mannitol used as a control treatment to control for osmolarity had no effect on the expression of pyroptosis related proteins including NLRP3, cleaved caspase-1 and N-terminal of GSDMD (Supplementary Figure S1). These results demonstrate that SAA could alleviate high glucose-induced pyroptosis by inhibiting the activation of NLRP3 inflammasome in HUVECs.

### Salvianolic acid A down-regulated PKM2/PKR pathway in high glucose-stimulated HUVECs

**FIGURE 6:**
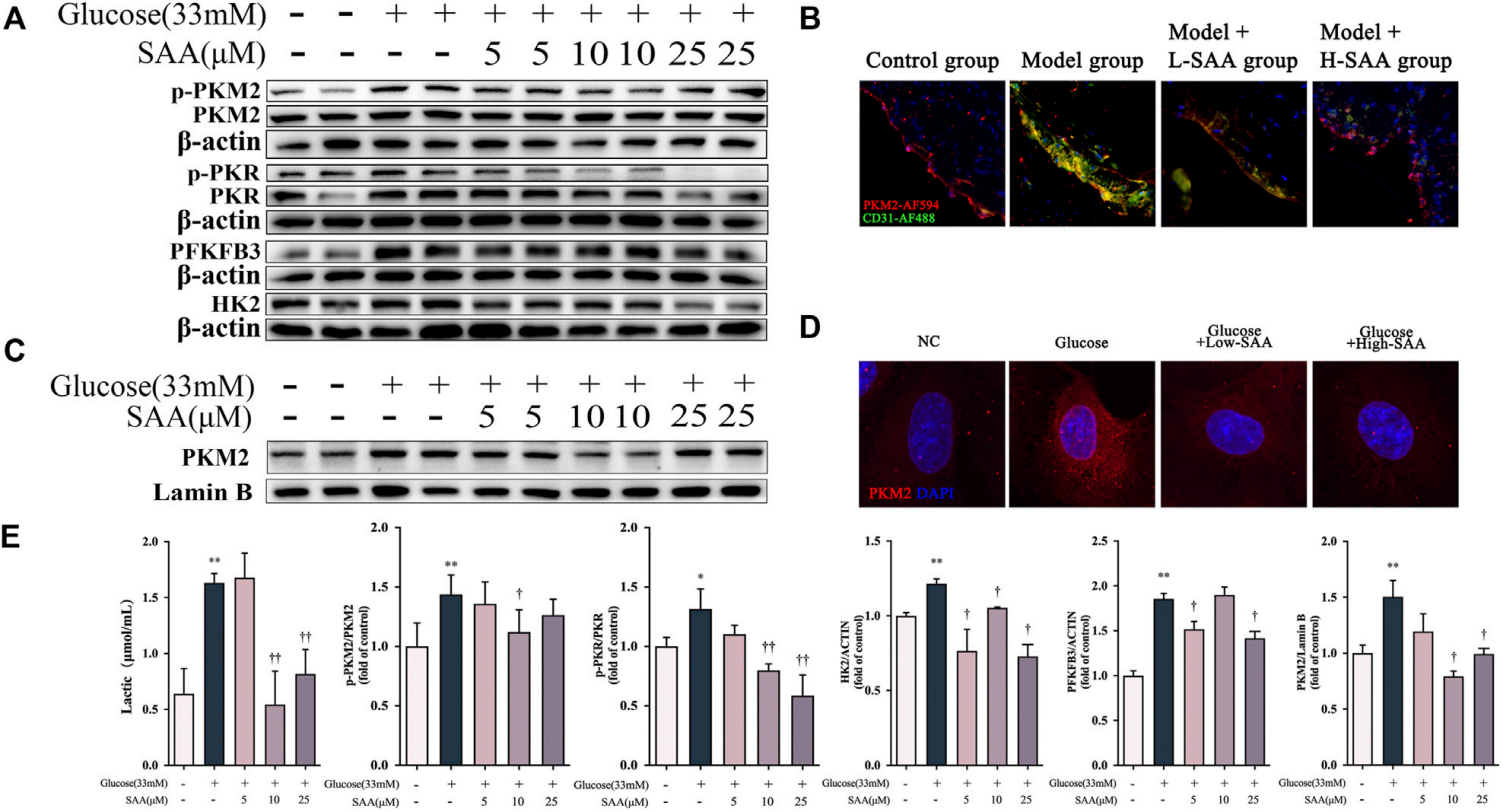
SAA Down-regulated PKM2/PKR pathway in high glucose-stimulated HUVECs. **(A)** Expression levels of pPKM2, pPKR, PKM2, PKR, HK2, PFKFB3 and *ß*-Actin in HUVECs were determined by western blot. **(B)** pPKM2 and CD31 levels in aortic roots were detected by immunofluorescence **(C)** Cellular localization of PKM2 was analyzed by immunofluorescence staining. **(D)** Nuclear fractions were isolated and PKM2 levels in HUVECs were detected by Western blotting. **(E)** Lactate levels in HUVECs. Data of western blot are the representatives of four independent experiments. Date of histograms are represented as mean ± SD of three independent experiments. *p < 0.05, **p < 0.01 vs. control group. ^†^ *p* < 0.05, ^††^ *p* < 0.01 vs. high glucose group.

Since PKR is a regulator of the NLRP3 inflammasome through autophosphorylation (Lu et al., 2012), we investigated whether the SAA influences pyroptosis *via* the regulation of PKR phosphorylation. The investigation revealed that the phosphorylation rate of PKR was increased in HUVECs exposed to 33 mM glucose, which was inhibited by SAA treatment in a dose-dependent manner (Figure 6A). Emerging evidence indicates that glycolysis activates the NLRP3 inflammasome in pyroptosis (Hughes and O’Neill, 2018). Additionally, it was reported that lactate treatment could induce phosphorylation of PKR and regulate activation of the NLRP3 inflammasome *via* lactate-dependent PKR phosphorylation (Lin et al., 2021). High glucose level was also found to induce overproduction of lactate (Figure 6E) and activate some key enzymes in glycolysis, including PFKFB3, LDH, and HK2 (Figure 6A). The results also demonstrate that SAA could inhibit the overproduction of lactate and overexpression of glycolysis-related proteins caused by high glucose levels than the DMSO used as a control. Furthermore, the results indicate that the phosphorylation level of PKM2, one of the important glycolysis-related proteins, at the Y105 site was enhanced by high glucose level in HUVECs despite no significant changes recorded in the level of its total protein expression (Figure 6A). Further treatment with SAA revealed that the rate of Y105 phosphorylation of PKM2 was decreased. Similar results were obtained from experiments on the endothelial cells of the aortic sinus (Figure 6B). Multiple findings suggest that Y105 phosphorylation of PKM2 is the major feature in the transition of PKM2 from a tetramer with high pyruvate kinase activity to a dimer with a low enzymatic activity which leads to subsequent nuclear translocation (Alquraishi et al., 2019). Nuclear translocation of PKM2 in the inflammatory response is considered the beginning of a positive feedback loop, whereby PKM2 localized in the nucleus acts as a transcription factor to drive the transcription of glycolysis-related and inflammatory genes (Alves-Filho and Pålsson-McDermott, 2016; Pan et al., 2022). Therefore, we examined if SAA protects HUVECs from pyroptosis by regulating the PKM2 shuttle between the cytosol and the nucleus. Induction of PKM2 nuclear translocation by 33 mM glucose, the images obtained from confocal microscopy, and the western blot analysis confirmed that SAA decreases the level of nuclear translocation (Figures 6C,D).

### Salvianolic acid A binds to PKM2 in HUVECs

**FIGURE 7:**
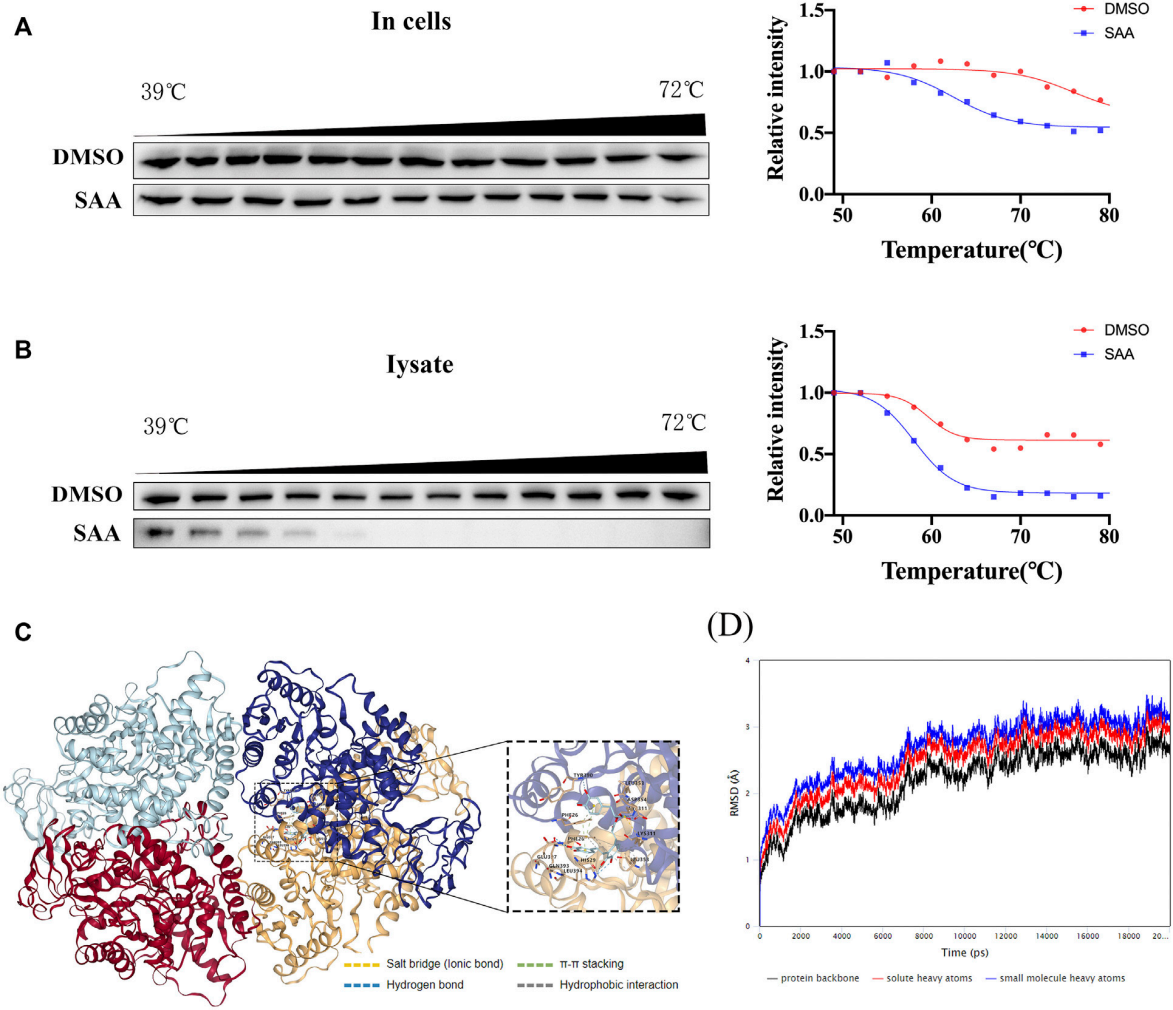
SAA binds to PKM2 in HUVECs. **(A–B)** Cells were incubated with DMSO or SAA for 6 h, and CETSA analyzed the thermal stabilization of PKM2 protein at different temperatures and concentrations (*n* = 3). **(C)** Binding pose of SAA and PKM2 as shown by molecular docking using AutoDock Vina. **(D)** Molecular dynamics simulation of PKM2-LBDs with SAA. Root-mean-square deviation (RMSD) and ligands for PKM2-LBDs in 20 ns molecular dynamics simulation.

Previous literature has reported that PKM2 plays a critical role in balancing glycolysis and inflammation through structural transformation. Thus, the interaction of SAA and PKM2 (Alves-Filho and Pålsson-McDermott, 2016) was investigated in this study. CETSA analyzes ligand-target binding by immunoblotting to determine the level of stability of target proteins against heat-induced precipitation (Molina et al., 2013). CETSA demonstrated that SAA significantly altered the thermal stability of PKM2 compared to the control group (DMSO treatment), further supporting the direct binding of SAA to PKM2 in the HUVECs lysates and intact HUVECs (Figures 7A,B). Next, a molecular docking analysis demonstrated that SAA probably binds to the activator pocket of PKM2 (PDB ID:1T5A) at PHE26, HIS29, LYS311, ASP354, LEU353, TYR390, GLN393, LEU394, and GLU397 (Figure 7C). To further verify the binding of SAA to PKM2, MDS was conducted to assess the stability of PKM2-LBDs binding with ligands (Figure 7D). After 20 ns of MDS, the SAA-to-PKM2-LBDs systems reached relatively stable states (Figure 7D). The results suggest that SAA might interact with PKM2, the key regulator of immunometabolic reprogramming.

### Salvianolic acid A protected the endothelial cells against high glucose-induced pyroptosis in a PKM2/PKR-dependent manner

**FIGURE 8:**
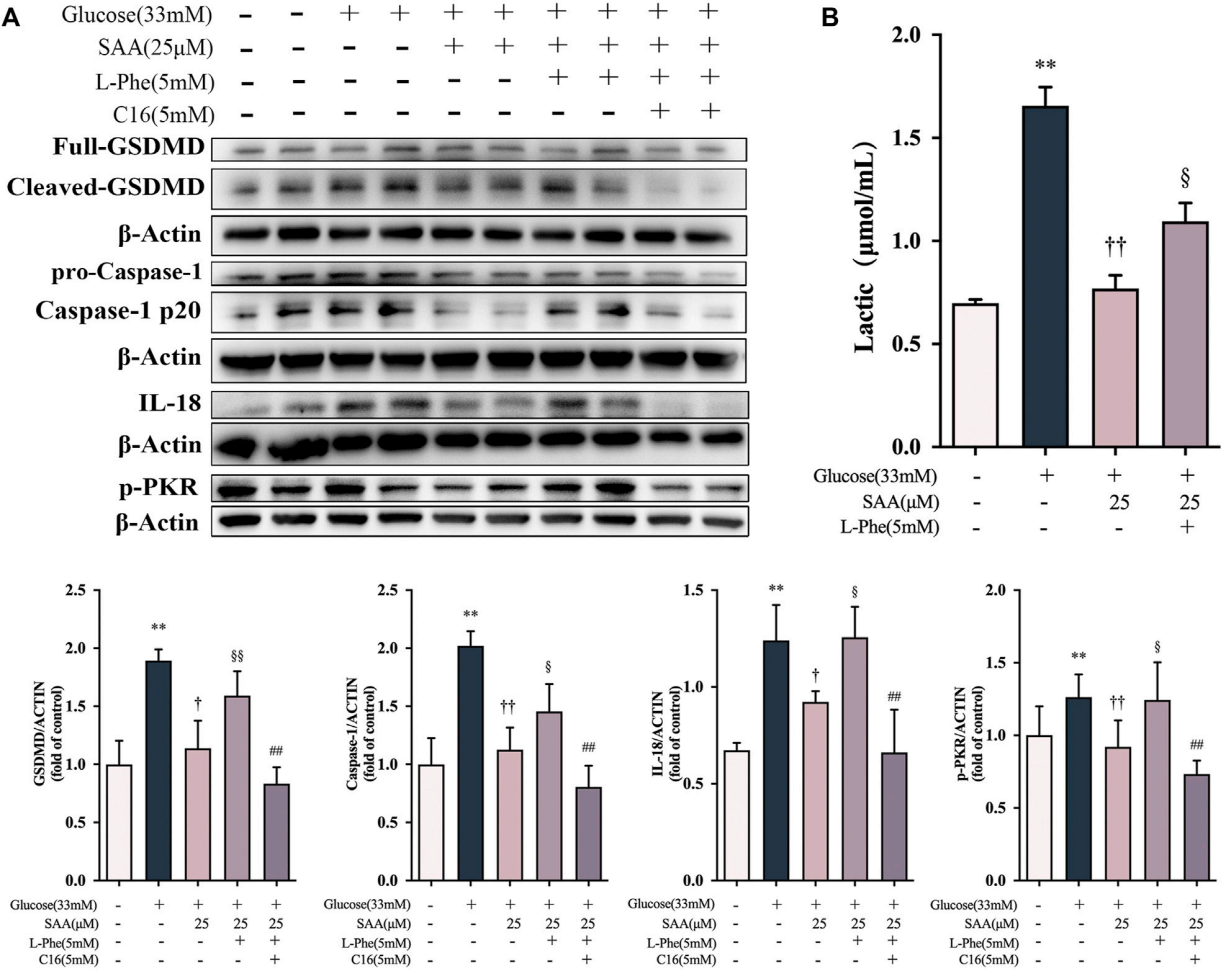
SAA protected the endothelial cells against high glucose-induced pyroptosis in a PKM2/PKR-dependent manner. **(A)** Expression levels of caspase-1, GSDMD, IL-18, and *ß*-Actin in HUVECs were determined by western blot. **(B)** Expression levels of pPKR, PKR and *ß*-Actin in HUVECs were determined by western blot. **(C)** Lactate levels in HUVECs. Data of western blot are the representatives of four independent experiments. Date of histograms are represented as mean ± SD of three independent experiments. *p < 0.05, **p < 0.01 vs. control group. ^†^ *p* < 0.05, ^††^ *p* < 0.01 vs. high glucose group. ^§^ *p* < 0.05, ^§§^ *p* < 0.01 vs. high glucose + SAA group. ^#^ *p* < 0.05, ^##^ *p* < 0.01 vs. high glucose + SAA + PHE group.

Based on our findings on the down-regulation of Y105 phosphorylation of PKM2 by SAA through direct binding with PKM2, we further investigated the potential relationship between the inhibitory effect of SAA on pyroptosis and PKM2. Phenylalanine (PHE) is a small molecule that binds to PKM2, resulting in structural alteration and inhibition of enzymatic activity (Morgan et al., 2013). Notably, pretreatment with PHE disrupted the inhibition of pyroptosis-related marker proteins such as the cleaved caspase-1 and GSDMD-N by SAA in the HUVECs exposed to 33 mM glucose (Figure 8A). The recovery of the SAA-suppressed IL-18 by PHE treatment has further corroborated the finding (Figure 8A). Pretreatment with PHE was also found to increase lactate production (Figure 8C) in HUVECs following SAA treatment, as previously reported (Yuan et al., 2018). The results demonstrate that PHE spiked the levels of SAA suppressed-PKR phosphorylation (Figure 8A). Additionally, a potent inhibitor of PKR kinase activity called C16 (Tronel et al., 2014) inhibited PHE-recovered pyroptosis-related marker proteins and production of IL-18 in high glucose-exposed SAA-treated HUVECs (Figure 8A), suggesting that SAA protects the endothelial cells against high glucose-induced pyroptosis in a PKR-dependent manner. These results demonstrate that the modulatory effect of SAA on high glucose-induced pyroptosis is partly mediated by PKM2-dependent PKR phosphorylation during inflammasome activation in HUVECs.

## Discussion

Due to the essential role of the endothelium in diabetic cardiovascular disease complications such as atherosclerosis, targeting the core events in their function might be a good strategy for therapeutic intervention. Despite the established protective effects of SAA on cardiovascular disease, the molecular mechanisms underlying the effect remains to be elucidated. This study is the first to report the role of SAA in inhibiting inflammasome activation in the aortic sinus and mitigating endothelial pyroptosis, which could potentially balance the stability of plaques and improve the health conditions related to diabetic atherosclerosis. These results suggest that SAA exerts some anti-atherosclerotic effects in the context of diabetes.

Atherosclerosis has been considered an inflammation-related disease (Engelen et al., 2022). In contrast, pyroptosis is a unique form of inflammatory cell death mediated by the inflammasome, which is dependent on caspase-1 activation. Pyroptosis has been associated with the release of inflammatory factors such as IL-1β and IL-18 when the N-terminus of GSDMD disrupt cell membranes. Recent studies have reported that the malfunction of various cells associated with pyroptosis involves the accretion of atherosclerotic plaque, suggesting a critical role of pyroptosis in atherosclerosis (He et al., 2021a; He et al., 2021b). Furthermore, the vascular endothelium at the site of inflammation is both an active participant and a regulator of the inflammatory process (Rowlands et al., 2011). This study verifies the involvement of inflammation pathways in the SAA treatment of diabetic atherosclerosis. Notably, emerging literature indicated that at early and advanced atherosclerosis stages, vascular endothelial pyroptosis is critically involved in the formation and destabilization of atherosclerotic plaques (Zhang et al., 2015; Kurdi et al., 2018; An et al., 2019).

Similarly, our investigation of diabetic atherosclerosis of ApoE^−/−^ mice revealed the enhanced expression level of NLRP3-ASC and GSDMD in the atherosclerotic plaques of endothelial cells with aortic sinus. However, data from the current study support the hypothesis that the modulation of endothelial pyroptosis involves the inhibition of atherosclerosis by SAA in ApoE^−/−^ mice with STZ-induced diabetes. Considering the relationship between SAA and the modulation of the NLRP3 inflammasome pathway, we evaluated whether the latter is involved in inhibiting HUVECs pyroptosis by SAA *in vitro*. The evaluation revealed that the expression of NLRP3, ASC, Caspase-1 p20, and GSDMD-NT was upregulated in high glucose-exposed HUVECs. In contrast, when treated with SAA, the endothelial cells became less responsive to high glucose-induced activation of pyroptotic death-related cascade procedures, suggesting that NLRP3 inflammasome and pyroptotic death are suppressed in the SAA treatment of HUVEC exposed to high glucose level.

This study reports the diverse mechanisms of endothelial pyroptosis and the involvement of classic NLRP3-ASC-Caspase1 inflammasome, GSDMD, and IL-18 signals. Despite the suggested regulatory role of SAA in the primary pathways, the findings are insufficient to elucidate the mechanism of SAA in relieving endothelial pyroptosis under diabetic conditions. A few recent studies have suggested the role of different metabolic conditions in influencing the functions and survival of endothelial cells (Li et al., 2019a). Several recent studies demonstrate the association between glycolysis and the activation of NLRP3 inflammasome and the execution of pyroptosis programming (Wen et al., 2012). Glycolysis and OXPHOS levels regulate NLRP3 inflammasome activation in macrophages (Lin et al., 2020). The production of mtROS regulated by hexokinase (HK) is believed to be important for glycolysis-dependent NLRP3 inflammasome activation (Moon et al., 2015).

Additionally, phosphorylation of PKR is required for NLRP3 inflammasome activation (Lu et al., 2012), whereby PKR physically interacts with NLRP3, NLRP1, NLRC4, or AIM2, which are each involved in inflammasome assembly and activation. Some studies have depicted that glycolytic metabolites, including lactate, may promote PKR phosphorylation and subsequent activation of NLRP3 inflammasome (Lu et al., 2012; Xie et al., 2016) in macrophages. Furthermore, activation of the NLRP3 inflammasome requires lactate-dependent PKR phosphorylation regulated by pyruvate kinase muscle isoenzyme 2 (PKM2) (Xie et al., 2016). Endothelial cells are “glycolysis-addicted” cells, and glucose metabolism in endothelial cells remains largely dependent on glycolysis even under adequate oxygen conditions (Leung and Shi, 2022). Such a phenomenon is associated with the need for endothelial cells as the first layer of cells in vascular tissue to transport oxygen to other cells and to carry out functions such as vascular regeneration in case of ischemia (Eelen et al., 2018). Despite the dependence of endothelial cells on glycolysis, maintaining mitochondria and the respiratory chain remains important, which is essential to maintain endothelial cells’ redox level and functional state. Therefore, it is vital to maintain a moderate and normal level of glycolysis for the function of endothelial cells (Rohlenova et al., 2018). Furthermore, excessive levels of glycolytic markers, including PFKFB3, HK2, and other key glycolytic proteins, as well as overproduction of lactate, have been reported in vascular endothelial cells inflicted by atherosclerosis, diabetes mellitus, and other vascular lesion-related diseases (Yang et al., 2018; Li et al., 2019b; Cao et al., 2019). We found that high glucose level has caused overexpression of several glycolysis-related proteins in HUVECs, accompanied by excessive lactate production, as reported in previous studies. Interestingly, several members of the salvianolic acid family also exhibit modulatory effects on glycolytic dysfunction. It has been elegantly demonstrated that salvianolic acid C and suppression of PFKFB3-driven glycolysis restrains endothelial-to-mesenchymal (EndMT) transition in endothelial cells (Zeng et al., 2022). And salvianolic acid B regulates macrophage polarization in ischemic/reperfused hearts by inhibiting mTORC1-induced glycolysis (Zhao et al., 2020). In the current study, SAA also showed a modulatory effect on the abnormal glycolysis-related indicators induced by high glucose. Additionally, a key enzymes in glycolysis called PKM2, was increased in the vascular endothelial cells with aortic sinus of Western diet-fed diabetic ApoE^−/−^ mice and in HUVECs exposed to high glucose levels. However, treatment with SAA was found to alleviate the level of PKM2 in both samples. PKM2 is increasingly recognized as the key component that connects the metabolic and inflammatory responses in different diseases, particularly atherosclerosis (Shirai et al., 2016; Pan et al., 2022). Several studies have reported a high expression level of PKM2 in atherosclerotic plaques (Shirai et al., 2016; Lü et al., 2018).

PKM2 is a specific protein in tetrameric and dimeric/monomeric forms (Chaneton et al., 2012). The tetrameric PKM2 has a high pyruvate kinase activity similar to PKM1, which catalyzes the production of pyruvate involved in the TCA cycle and maintains the flow of the glycolytic process. The dimeric/monomeric PKM2 has a lower enzymatic activity and is primarily involved in the translocation to the nucleus (Dombrauckas et al., 2005). Thus, PKM2 is not only a key enzyme in glycolysis but also carries out moonlighting functions that allow it to enter the nucleus, as demonstrated by growing evidence gathered from different biological contexts (Alquraishi et al., 2019). In addition, the role of PKM2 in immunometabolic reprogramming in the inflammatory response has been increasingly recognized (Alves-Filho and Pålsson-McDermott, 2016; Liu et al., 2021). PKM2 has exhibited superiority as a potential intervention target in several inflammatory physiological processes and diseases (Alves-Filho and Pålsson-McDermott, 2016). Furthermore, PKM2 has been reported to be the subject of multiple posttranslational modifications, such as phosphorylation, acetylation, cysteine redox modifications, and S-nitrosylation, which typically result in conformational changes and subsequent functional alterations (Zheng et al., 2021).

Phosphorylation of PKM2 at the Y105 site has been frequently associated with the activation of inflammatory responses, which inhibits the formation of tetrameric PKM2, leading to the accumulation of monomeric/dimeric PKM2. PKM2 in monomeric/dimeric forms further gains entry into the nucleus and initiates the Warburg effect (Hitosugi et al., 2009; Palsson-McDermott et al., 2015; Xu et al., 2020b). The loss of pyruvate kinase activity and the nucleation of PKM2 has been identified as the initiators of the positive feedback loop that drives the glycolytic-inflammatory response—in the absence of PKM2 pyruvate kinase activity, overproduction of lactate occurs, and the nucleation of PKM2 acting as a transcription factor not only drives the transcription of inflammation-related genes but also triggers the transcription of glycolysis-related genes such as LDHA, further increases the production of lactate (Palsson-McDermott et al., 2015; Alves-Filho and Pålsson-McDermott, 2016). Furthermore, the loop continues to involve PKM2 since several metabolites influence the level of PKM2 expression and the upregulation of the associated regulatory genes (Pan et al., 2022). We have also found that nuclear localization of PKM2 was increased in HUVECs exposed to high glucose levels, while SAA treatment has reduced the localized content of PKM2 in the nucleus. This alteration is consistent with the reduced phosphorylation PKM2 at the Y105 site, the decrease in the expression level of glycolysis-related proteins, and reduced lactate production by SAA. Notably, PKM2 has been identified as the major isoform of pyruvate kinase in endothelial cells, which is important for regulating energy metabolism, proliferation migration, and inflammatory response. Therefore, inhibition of PKM2 expression by pharmacological or molecular biological risks the potential alteration in endothelial cells’ normal physiological function and survival (Kim et al., 2018; Stone et al., 2018). The structure of PKM2 is complex, and a series of small molecules have been developed to improve its structural stability and enzymatic activity (Anastasiou et al., 2012; Lu et al., 2021). Through molecular docking, we have identified several active sites of PKM2 that SAA may bind to PHE26, HIS29, LYS311, ASP354, LEU353, TYR390, GLN393, LEU394, and GLU397 through salt bridge interactions, hydrogen bond interactions, π-π stacking interactions, and hydrophobic interactions. These binding sites are in the activator pocket of PKM2 (Li et al., 2021b), which shares a very similar binding mode with quinoline sulfonamides (a compound that has been reported to promote tetramer formation of PKM2) (Kung et al., 2012). Additionally, MDS demonstrated that the SAA-to-PKM2-LBDs systems are relatively stable. Also, the results obtained from CETSA support the potential direct binding of SAA to PKM2 in the cells. It was also found that the binding to the active site of PKM2 could increase its pyruvate kinase activity.

Recently, there has been an increasing interest in the research to elucidate the link between PKM2 and activation of inflammasomes/pyroptosis and to investigate the role of PKM2 as a potential therapeutic target in treating diseases caused by inflammasomes/pyroptosis. Several studies have demonstrated the partial link between PKM2 and pyroptosis in metabolisms of diseases (Li et al., 2020, Li et al., 2021a). We have conducted a preliminary investigation to identify the potential association between PKM2 and pyroptosis of endothelial cells exposed to high glucose levels. PHE, a modifier that inhibits PKM2 activity (Morgan et al., 2013; Yuan et al., 2018; Macpherson et al., 2019), was found to inhibit the inhibition activity of SAA on high glucose-induced pyroptosis, matured inflammatory factors, and phosphorylated PKR in HUVECs, as previously reported (Yuan et al., 2018), which restored glycolysis-related protein expression and overproduction of lactate. PKM2 remains an attractive target in regulating endothelial cell injury in metabolic and inflammatory diseases. In addition, the strategy for drug development needs to be revised, and consideration should also be given to the use of glycolytic or PKM2-inhibiting class interventions, taking into account the dependence of endothelial cells on PKM2. As expected, C16, a potent inhibitor of PKR kinase activity (Tronel et al., 2014), inhibited PHE-recovered pyroptosis-related marker proteins and IL-18 production in SAA-treated HUVECs exposed to high glucose levels. Therefore, it may be deduced that improvement of diabetic atherosclerotic lesions *in vivo* by SAA is mainly due to the regulation of aorta pyroptosis of endothelial cells *via* targeting PKM2 and consequent PKR phosphorylation and activation of NLRP3 inflammasome.

In conclusion, we have revealed a novel function of SAA in inhibiting pyroptosis in endothelial cells and Western diet-fed STZ-induced mice with diabetic atherosclerosis. The findings provide new insights into the NLRP3 inflammasome activation and pyroptosis in vascular endothelium, which is important for studying diabetes-related atherosclerotic coronary artery disease progression and therapy. The action of SAA on PKM2 results in the inhibition of lactate-dependent PKR phosphorylation and NLRP3 inflammasome activation, alleviating endothelial pyroptosis and offering a novel therapeutic approach for treating diabetic atherosclerosis.

## Data Availability

The original contributions presented in the study are included in the article/Supplementary Material, further inquiries can be directed to the corresponding authors.
