# Supplementary figures and images for: Salvianolic acid A regulates pyroptosis of endothelial cells via directly targeting PKM2 and ameliorates diabetic atherosclerosis
Source: Front Pharmacol. 2022 Nov 8;13:1009229. doi: 10.3389/fphar.2022.1009229 (PMC9679534; doi:10.3389/fphar.2022.1009229)

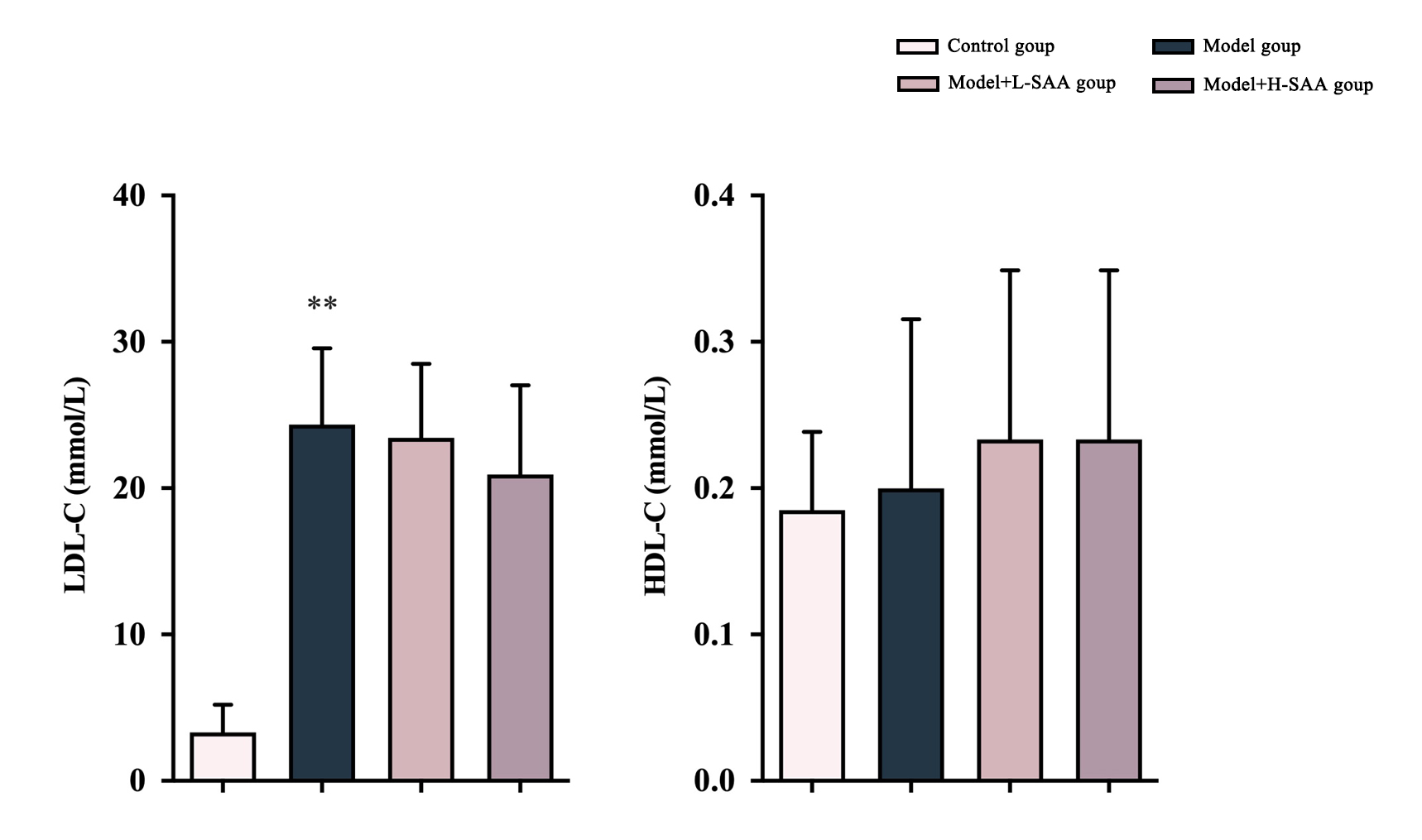

Supplement: Supplementary file 1 [file Image3.JPEG]

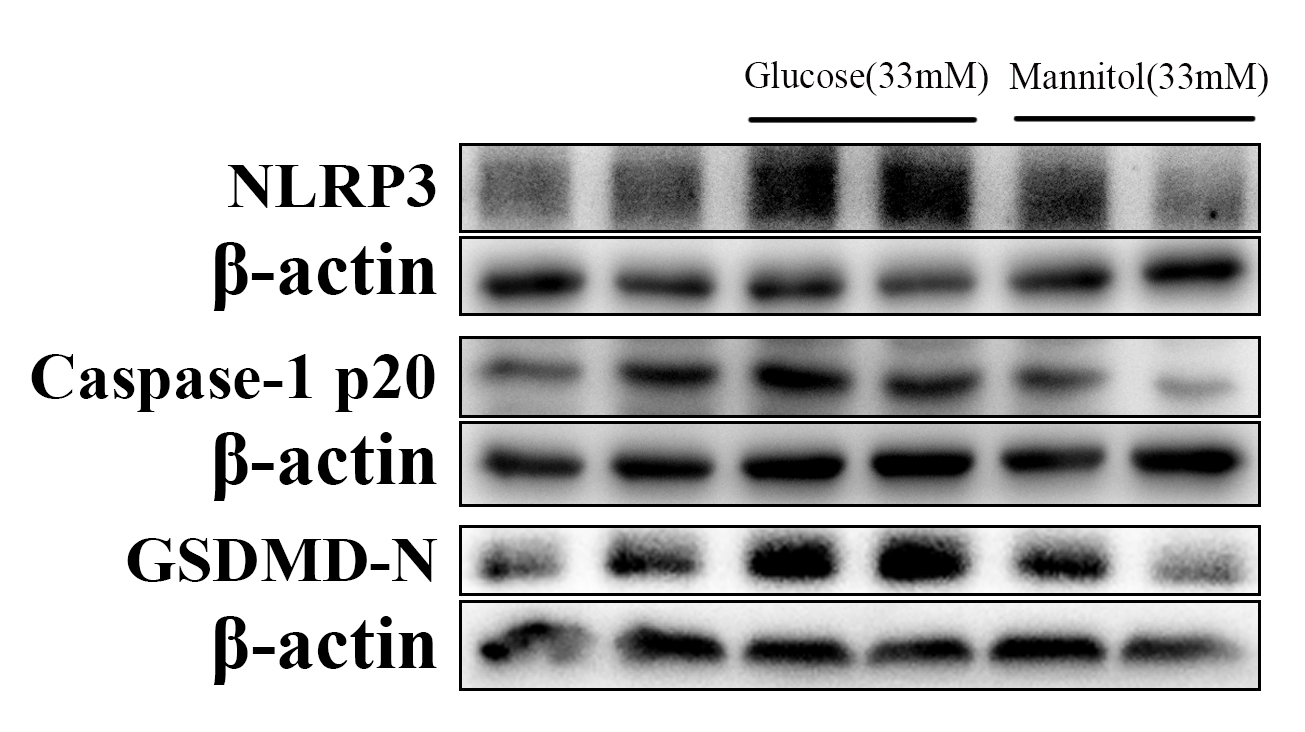

Supplement: Supplementary file 2 [file Image1.JPEG]

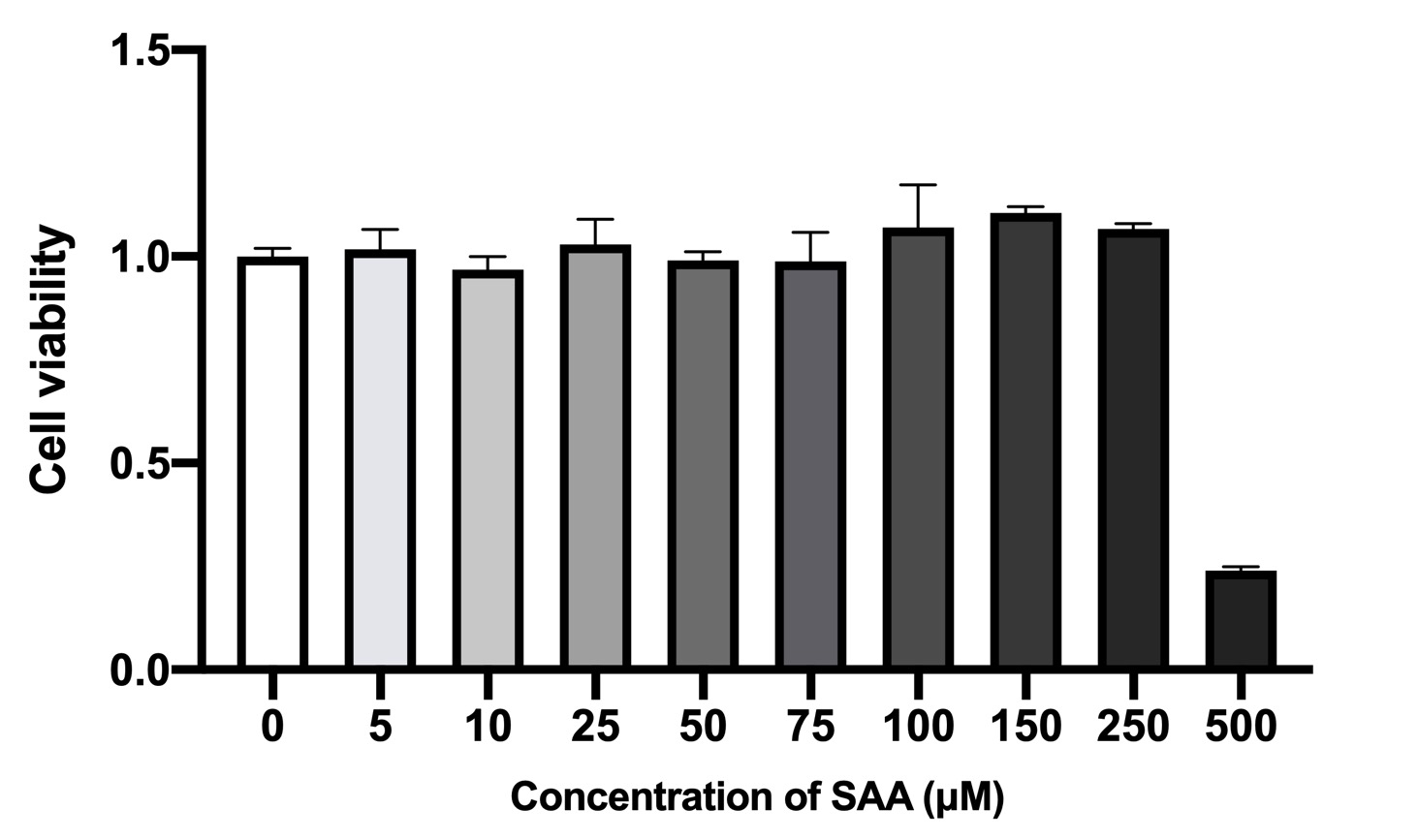

Supplement: Supplementary file 3 [file Image2.JPEG]
