# Supplementary material for: Salvianolic acid A regulates pyroptosis of endothelial cells via directly targeting PKM2 and ameliorates diabetic atherosclerosis
Source: Front Pharmacol. 2022 Nov 8;13:1009229. doi: 10.3389/fphar.2022.1009229 (PMC9679534; doi:10.3389/fphar.2022.1009229)
Supplement: Supplementary file 4 [file DataSheet1.docx]

Supplementary Figure1.


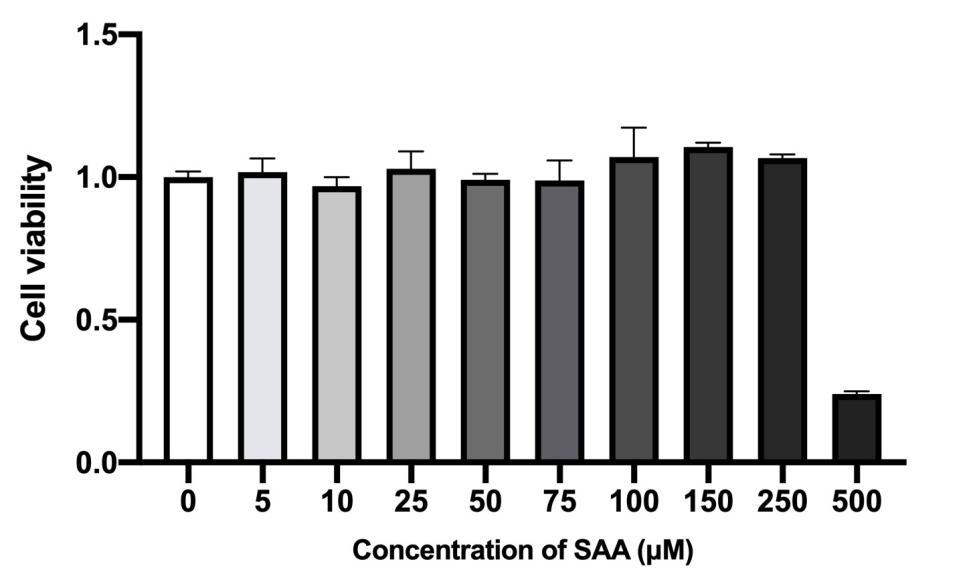


The effect of SAA on the cell viability of HUVECs in vitro.


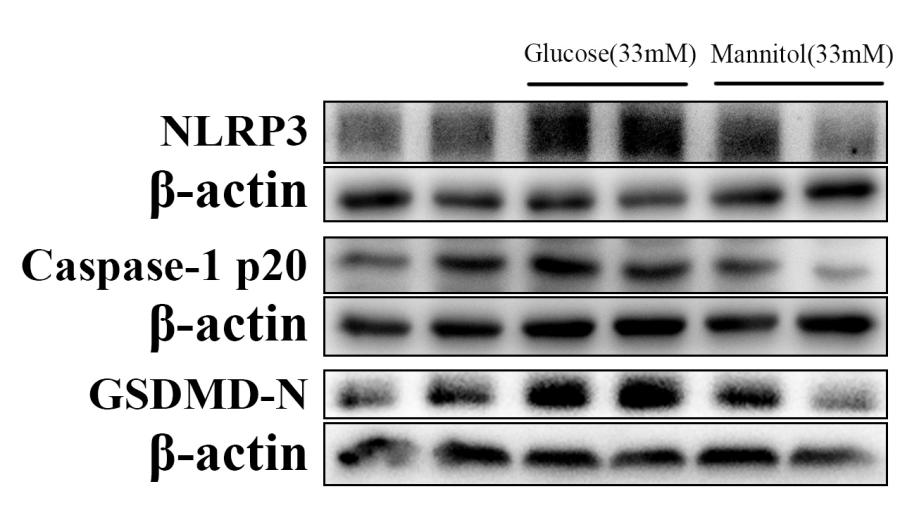


HUVECs cultured in different media containing normal glucose (5.5 mM), high glucose (33 mM), and mannitol (33 mM) was used as the osmotic control for HG. Expression levels of NLRP3, ASC, caspase-1, GSDMD and β-Actin in HUVECs were determined by western blot.
